# Cancer cachexia: Pathophysiology and association with cancer-related pain

**DOI:** 10.3389/fpain.2022.971295

**Published:** 2022-08-22

**Authors:** Michelle L. Law

**Affiliations:** Department of Human Sciences and Design, Robbins College of Health and Human Sciences, Baylor University, Waco, TX, United States

**Keywords:** cancer, inflammation, pain, cachexia, fatigue, anorexia, nutrition impact symptoms, muscle atrophy

## Abstract

Cachexia is a syndrome of unintentional body weight loss and muscle wasting occurring in 30% of all cancer patients. Patients with cancers most commonly leading to brain metastases have a risk for cachexia development between 20 and 80%. Cachexia causes severe weakness and fatigue and negatively impacts quality and length of life. The negative energy balance in cachectic patients is most often caused by a combination of increased energy expenditure and decreased energy intake. Basal metabolic rate may be elevated due to tumor secreted factors and a systemic inflammatory response leading to inefficiency in energy production pathways and increased energy demand by the tumor and host tissues. A growing body of research explores physiological and molecular mechanisms of metabolic dysregulation in cachexia. However, decreased energy intake and physical functioning also remain important contributors to cachexia pathogenesis. Pain associated with metastatic malignancy is significantly associated with inflammation, thus making inflammation a common link between cancer pain and cachexia. Pain may also influence appetite and food intake and exacerbate fatigue and functional decline, potentially contributing to cachexia severity. Cancer pain and cachexia often occur simultaneously; however, causal relationships remain to be established. Appropriate assessment and treatment of pain in advanced cancer patients may positively impact nutrition status and physical functioning, slowing the progression of cachexia and improving quality and length of life for patients.

## Introduction

Cachexia is a syndrome of progressive and unintentional weight loss and muscle atrophy that occurs in multiple inflammatory disease states such as heart failure, kidney failure, HIV/AIDS, and cancer (1). Cachexia occurs in a large percentage of advanced cancer patients (1). Patients with cancers of the gastrointestinal tract, lung, and liver have a risk for developing cachexia between 70 and 90% (2). Although cachexia is relatively uncommon in patients with primary brain tumors, patients with tumors commonly leading to secondary brain metastases, including lung, breast, and skin (3), have a cachexia risk between 20 and 80% (2).

Importantly, cachexia diminishes both quality and length of life. Patients with cachexia experience severe weakness and fatigue with impaired physical functioning (1), increased chemotherapy toxicity (4) and decreased efficacy (5), and greater risk of post-surgical complications (6). Cachexia also significantly increases medical costs (6, 7).

Cancer cachexia is divided into three stages – pre-cachexia, cachexia, and refractory cachexia (8). Pre-cachexia is defined as weight loss ≤ 5% with the presence of anorexia or metabolic abnormalities such as glucose intolerance. Cachexia is defined as body weight loss of >5%, or >2% in patients with a BMI <20 kg/m^2^ or the presence of sarcopenia. Refractory cachexia occurs when patients are in a highly catabolic state with a life expectancy of <3 months (8). The progression of cachexia is variable and can be influenced by tumor type, cancer progression, food intake, severity of inflammation, and response to anti-cancer therapies (8).

The characteristic muscle wasting occurring in cachexia is not fully reversible by nutrition support alone, necessitating a multi-modal and multi-disciplinary approach to treatment (9). Identification of and intervention for patients with cachexia may improve symptoms and treatment outcomes (10). Interventions currently involve nutrition and physical therapy, and treatment of underlying causes of decreased food intake such as anorexia and nausea (11). These treatments generally do not reverse cachexia but may slow its progression. Amorelin, a ghrelin-agonist, was recently approved for cancer cachexia treatment in Japan (12), but no approved pharmaceutical therapies are yet available in the United States and Europe. Furthermore, similar to nutrition and physical therapy, amorelin may decrease the severity of cachexia, but does not cure this progressive syndrome.

The aim of this review is to provide an overview of cancer cachexia pathophysiology and review evidence of the association between cancer-related pain and cachexia.

## Cancer cachexia pathophysiology

Cancer cachexia is a multi-organ syndrome of systemic inflammation and negative energy balance. Inflammation originates from both tumor and host tissue, with tumor-secreted cytokines activating host immune cell cytokine production. Inflammatory cytokines induce signaling in the central nervous system (CNS) and peripheral tissues to alter metabolism, increasing catabolic and decreasing anabolic signaling. The involvement of inflammation in cachexia has been extensively reviewed (1, 13–15), and is associated with cancer-related weight loss in humans (16). Additional non-inflammatory mediators including activin A, myostatin, GDF15, and lipocalin-2, have also been implicated in catabolic signaling in cachexia (17). Although skeletal muscle is currently the major focus of cachexia research because of the severe wasting occurring in this tissue, other tissues such as cardiac muscle, liver, bone, the gastrointestinal tract, and adipose tissue are also affected by and contribute to cachexia pathology *via* numerous crosstalk mechanisms (13). Ultimately, weight loss and muscle wasting in cachexia occur because of significant energy imbalance in the presence of an inflammatory environment. Most often, negative energy balance results from both increased energy expenditure and decreased energy intake (1).

### Increased energy expenditure

**Figure 1:**
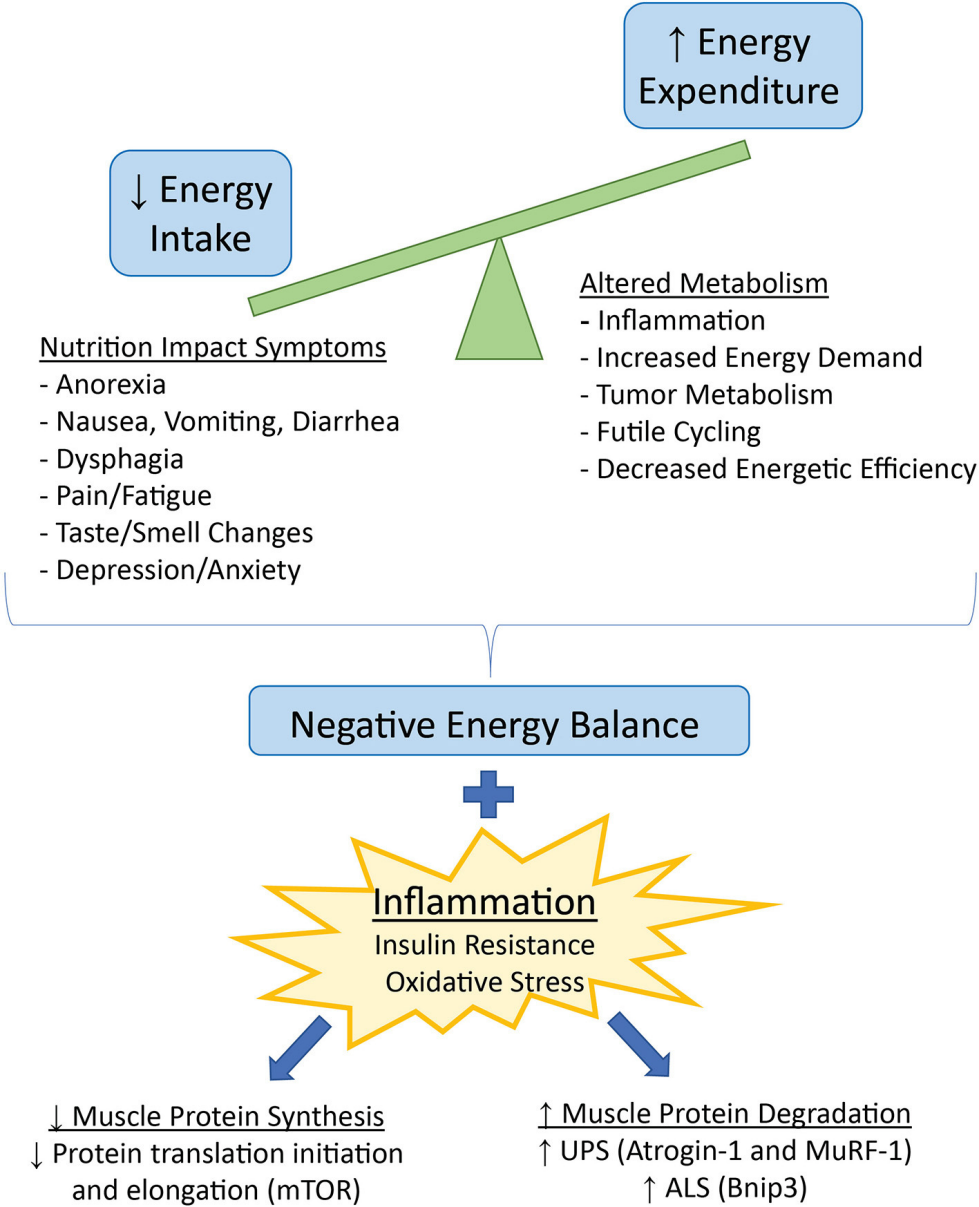
Factors contributing to decreased energy intake and increased energy expenditure, leading to a net negative energy balance and weight loss in cancer cachexia. Negative energy balance in the presence of inflammation and related insulin resistance and oxidative stress favors loss of skeletal muscle mass *via* downregulation of protein synthesis and upregulation of protein degradation. UPS, ubiquitin-proteasome system, ALS, autophagy lysosome system.

Resting energy expenditure (REE), measured by indirect calorimetry, was increased in two studies of treatment-naïve cancer patients both with and without weight loss, suggesting elevated REE may contribute to both cachexia development and progression (18, 19). Causes of increased REE are multifactorial and include increased immune response and inflammation, increased energy demand by metabolically active organs, decreased energetic efficiency, and competition for energy substrates by the tumor (Figure 1). Pancreatic cancer patients with increased host immune response, measured by serum c-reactive protein (CRP) and isolated peripheral blood mononuclear cell cytokine production, had a higher REE than patients without an acute-phase response (20). Increased relative mass of the liver, a metabolically demanding organ (21), was correlated with increased REE in colorectal patients, and was estimated to cumulatively increase REE by over 17,000 kcal during the last 3 months of life (22). Increased heart rate, a predictor for mortality in cancer patients (23, 24), was positively associated with REE (19). Treatment of cancer patients with a beta-adrenergic receptor antagonist decreased both heart rate and REE, implicating sympathetic nervous system activation as a contributor to increased energy demands (25, 26). Adipose tissue browning may also increase REE. Increased browning of adipose has been identified in human cancer patients (27, 28), but most research on mechanisms of adipose tissue browning in cachexia has been done with animal models (28–30). Brown adipose tissue expresses uncoupling protein 1 (UCP1), which uncouples electron transport from ATP synthesis, instead producing heat. Tumor burden can also contribute to energy demands. In addition to the mass of the tumor, the rate of glycolysis in tumor cells contributes to the total energy requirements of the tumor. Many tumors exhibit increased anaerobic metabolism, leading to increased glucose uptake, glycolysis, and lactate production (31). Lactate production by the tumor increases hepatic gluconeogenesis *via* the Cori cycle and subsequently decreases energetic efficiency by the tumor cells (32).

### Decreased energy intake

In addition to increased energy expenditure, many patients with cancer cachexia also have significantly reduced energy intake caused by a variety of Nutritional Impact Symptoms (NIS), any of a broad range of symptoms impeding nutrient intake (33) (Figure 1). The tumor stage and location, and the type of anti-cancer therapy prescribed can influence the type and amount of food consumed. Tumors that originate from or have metastasized to the head and neck or gastrointestinal tract can cause dysphagia, early satiety, and malabsorption of nutrients. Chemotherapy and radiation can cause decreased appetite due to altered taste and smell, food aversions, nausea, vomiting, and diarrhea (34, 35). Decreased appetite may also occur as a direct result of inflammation and altered hunger/satiety signaling in the hypothalamus (1). Psychosocial aspects of cancer are another important consideration in the development of cancer-induced weight loss (36). Furthermore, as discussed in the next section, pain and fatigue may decrease both appetite and food intake, as well as physical activity.

### Consequences of negative energy balance

Negative energy balance resulting from increased energy expenditure and decreased energy intake over time causes weight loss. In healthy individuals, intentional weight loss from reduced food intake and increased exercise favors loss of fat and preservation of muscle. In contrast, weight loss in a disease state such as cancer leads to significant loss of both fat and lean tissue, particularly skeletal muscle, due to the presence of non-inflammatory (17) and inflammatory (15) mediators. Inflammatory cytokines act to upregulate catabolic signaling (37, 38) and impair regeneration (39) in skeletal muscle, particularly through the transcription factor NF-kappaB. Inflammation in cancer cachexia has also been implicated in resistance to the anabolic hormone insulin (40, 41) altering substrate availability and utilization and increasing muscle protein breakdown. Finally, inflammation in cachexia is associated with mitochondrial dysfunction (42), which may decrease oxidative metabolism and increase oxidative stress in skeletal muscle (43) (Figure 1).

Catabolic signaling in a disease state such as cancer causes an imbalance between protein synthesis and protein degradation in skeletal muscle. Protein synthesis is decreased primarily through inhibition of various steps in the mTOR signaling pathway, inhibiting protein translation initiation and elongation (Figure 1). Indeed, mTORC1 signaling is blunted in cachectic mice (44), and ~60% decrease in protein synthesis has been measured in both cachectic rats (45) and mice (44). Protein degradation is increased *via* upregulation of two major pathways – the autophagy lysosomal system (ALS), and the ubiquitin-mediated proteolysis system (UPS) (Figure 1). The ALS enables macro-degradation of whole organelles or sections of cytosol, which is important in clearing damaged parts of the cell and improving cell survival (46). Markers of autophagy such as Bnip3 are consistently upregulated in experimental cachexia (40, 43); however, whether or not upregulation of the ALS is beneficial or detrimental in cachexia is unknown. Both insufficient and excessive autophagy can lead to cellular damage and death (47). The UPS is a targeted protein degradation system mediated by E3 ligase ubiquitin-tagging of proteins for proteolysis by the proteasome. Two major E3 ligases in the UPS, Atrogin-1 and MuRF-1, are implicated as necessary and sufficient to induce muscle wasting in cachexia *via* targeting of contractile proteins in the sarcomere (48, 49). Muscle-specific E3 ligases are transcriptionally upregulated by inflammatory cytokine signaling and activation of the NF-kappaB pathway (37), as well as *via* decreased insulin and insulin-like growth factor (IGF-1) signaling (50).

## A role for pain in cachexia development and progression

The International Association for the Study of Pain defines pain as “an unpleasant sensory and emotional experience associated with actual or potential tissue damage, or described in terms of such damage” (51). Pain is a common symptom in cancer patients, with nearly two-thirds of metastatic cancer patients experiencing cancer-related pain and over 75% of those patients reporting moderate to severe pain (52). Assessment of pain in cancer is challenging due to the many underlying causes of pain and numerous assessment methods used in the literature. Indeed, improved standardization of pain assessment in cancer patients may allow for better comparison of various pain therapies across multiple groups of people and multiple studies (53).

### Causes of cancer pain

**Figure 2:**
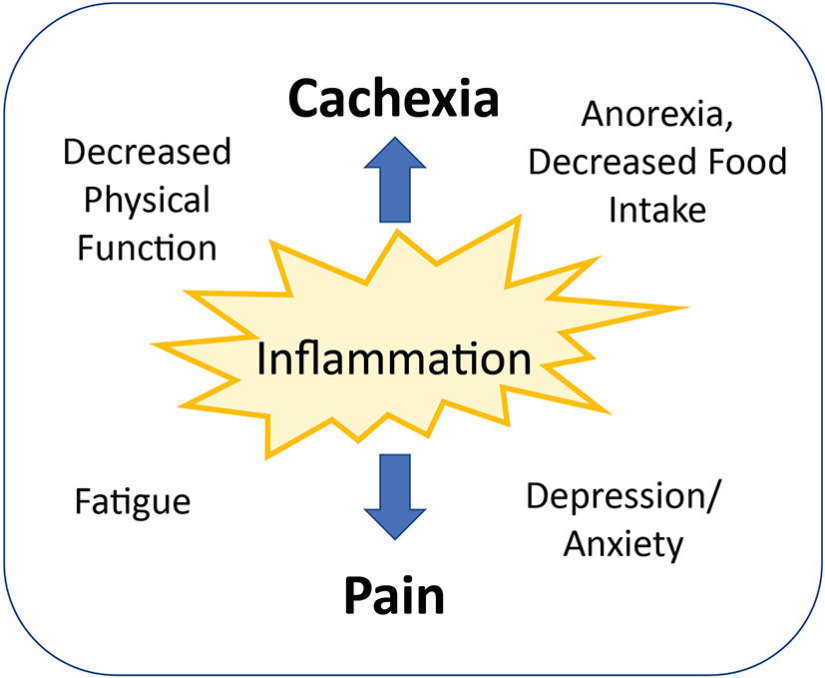
Inflammation is a common link between cancer-related pain and cachexia. Other mediating factors commonly occurring with both pain and cachexia include decreased physical functioning, increased fatigue, impaired food intake, and decreased mental health.

Cancer-related pain can be associated with the presence of the tumor in various places in the body, causing compression, invasion, or injury of organs and tissues. Ischemia, an acidic tumor microenvironment, and proteolytic enzyme secretion by tumors also may directly damage surrounding tissues and nerves (54). In addition to factors associated with the physical presence of the tumor, the inflammatory process occurring because of the tumor is likely responsible for a considerable proportion of cancer-related pain. Inflammatory cytokines may be produced by the tumor itself, but host immune cell activation also leads to significant upregulation of cytokine production, including TNF-alpha, IL-1beta, IL-6, EFG, TGF-beta, and PDGF (54). These cytokines may lead to pain by diffusing or being transported across the blood-brain barrier, or binding and activating primary afferent neurons (nociceptors) in the periphery (55). Cytokine signaling in the CNS has been implicated in cancer related symptoms including pain, fatigue, altered appetite, and depression/anxiety (54, 55). Supporting the proposed relationship between inflammation and pain is a retrospective analysis of two clinical trials including 718 cancer patients. Pain assessed at baseline *via* the pain subscale of the European Organization for Research and Treatment of Cancer Quality of Life Questionnaire C-30 (EORTC QLQ-C30) was significantly associated with C-reactive protein (CRP), a hepatic secretory protein associated with systemic inflammation (56). These findings were confirmed in a more recent study of 1,513 advanced cancer patients, which found patients in the highest quartile of CRP values had significantly increased pain compared to those in the lowest quartile (57). Moreover, another study identified high serum IL-6 concentrations in non-responders to the pain medication tramadol, which resulted in the use of stronger opioids in this group of patients (58). Interestingly, the prevalence of cachexia was also higher in non-responders, with cachexia being the only significant predictor of non-response after multivariate logistic regression analysis (59). Because inflammatory cytokines are involved in both the pain response and altered energy balance and metabolism found in cachexia, inflammation may be a common link between pain and cachexia, with other mediating factors being implicated in both conditions (Figure 2).

### Association between cancer pain and cachexia

There are numerous studies that seek to identify a relationship between cachexia or cachexia risk factors and various symptoms and complications arising from cancer. Due to the complexity and interrelatedness of many symptoms, cause-effect relationships are difficult to establish. Therefore, it is unknown whether pain contributes to cachexia, cachexia contributes to pain, or if pain and cachexia simply coexist as evidence of advancing disease. Most likely, all three may be true. Moreover, published studies to date generally include pain assessment as one of many measurements, making it challenging to determine independent effects of pain on cachexia. Regardless of limitations in existing literature, evidence points to an association between pain and cachexia in cancer patients. A cross-sectional study of 306 advanced cancer patients by Zhou et al. identified an association between cachexia severity (no cachexia, pre-cachexia, cachexia, refractory cachexia) and increasing symptom burden measured with the M.D. Anderson Symptom Inventory. Both pain and fatigue were significant predictors of increasing cachexia severity (60). A prospective study by Daly et al. categorized more than 1,000 cancer patients on a weight loss grading scale from 0 to 4, with scores based on current BMI and reported weight loss. Higher scores were associated with pain, fatigue, dyspnea, anorexia, and decreased quality of life (QoL) measured by the EORTC QLQ-C30 (61). These studies importantly establish a relationship between increasing cachexia severity and pain. Additional studies reviewed below identify potential mediating factors between pain and cachexia development. These factors include appetite, energy intake, weight loss, physical activity and performance, strength, lean body mass, and fatigue.

#### Pain and energy intake

Several studies identify a relationship between pain and decreased energy intake, a major contributor to negative energy balance in cachexia. In a multivariate logistic regression analysis of 368 treatment-naïve head and neck cancer patients, pain measured by the Head and Neck Patient Symptom Checklist (HNSC) was one of several significant predictors for decreased energy intake (62). Two studies have attempted to quantify the energy deficit associated with cancer-related pain. Bye et al. found pancreatic cancer patients with moderate to severe pain with movement, pain at rest, and pancreatic pain had a decreased energy intake between 600 and 1,500 kcal/day compared to patients with low intensity pain. Pain in this study was assessed by EORTC QLQ-C30 and the Edmonton Symptoms Assessment System (ESAS) and food intake assessed by 24-h recall (63). Kubrak et al. used generalized estimated equation (GEE) modeling to quantify the effect of pain on energy intake and weight loss in head and neck cancer patients. They found a 1 unit increase in pain (measured by a 5-point Likert scale) predicted for a decreased energy intake of 199 kcal/day and 1 kg weight loss in head and neck cancer patients receiving both chemo- and radiotherapy (64). Pain did not predict decreased energy intake in radiotherapy only patients, although a 1 unit increase in pain did predict for a 0.67 kg weight loss in this group, suggesting other factors besides energy intake are involved in weight loss (64).

#### Pain and activity/fatigue

Pain also predicts for decreased physical activity, performance status, and fatigue, which may contribute to cachexia by exacerbating muscle atrophy. Fouladiun and colleagues found patients with advanced cancer and cachexia had increased pain (outside the 99% CI) compared to normative values, measured by the SF-36. SF-36 scores for bodily pain and physical functioning predicted for decreased physical activity measured by an accelerometer (65). SF-36 bodily pain score was also significantly correlated with Karnofsky Performance Status in pancreatic cancer patients (66). In a secondary analysis of two clinical trials involving 654 patients, pain, depression, and fatigue were found to be clustered symptoms, occurring in combination 2–4 times more often than would be expected by chance. This cluster was significantly related to decreased physical functioning measured by the EORTC QLQ-C30 (67). Further evidence of the clustering of pain, depression, and fatigue is found in a subsequent study of 720 palliative care cancer patients, 44% of whom had “severe” levels of fatigue. Logistic regression identified pain and depression as independent predictors of fatigue in this population (68).

#### Pain and muscle mass/strength

A consequence of decreased energy intake and physical functioning is loss of lean mass and muscle strength, and these have also been associated with pain in cancer patients. Kilgour et al. found increased fatigue measured by the Brief Fatigue Inventory (BFI) to be related to decreased handgrip strength, quadriceps strength, and skeletal muscle mass index (SMMI) determined with DXA imaging in men with lung and gastrointestinal cancers. Interestingly, in multivariate regression analysis, pain, measured by ESAS, was a significant predictor in all three multivariate models (69), suggesting a potential link between pain and muscle mass and strength. Derksen et al. evaluated the effect of lean mass on EORTC QLQ-C30 symptoms in chemotherapy-treated CRC patients that had achieved stable disease or remission. Patients with increased lean mass had significantly reduced fatigue and pain compared to patients experiencing lean mass loss (70).

### Outcomes of pain and cachexia management

From the above discussion, there appears to be a strong association between cancer cachexia, mediators of cachexia, and cancer-related pain, although causal relationships remain difficult to establish. It seems plausible then, that pain management may improve cachexia and *vice versa*, but there is a scarcity of literature to examine this question. Pain is considered a nutrition impact symptom (NIS) (33), and multidisciplinary clinics designed to address NIS have reported both increased rates of symptom management (34) and decreased severity of pain, fatigue, and anorexia (71). However, in these studies, cachexia-related outcomes such as energy intake and body mass and composition were not measured. Limited evidence suggests management of cachexia-related symptoms may lead to improvements in pain. Although not broadly recommended for cachexia management (72), enteral nutrition support in a small cohort of pancreatic cancer patients led to increased BMI and lean mass, along with a trend toward improved pain and fatigue scores on the EORTC QLQ-C30 (73). The major limitation in this study was the high attrition, potentially leading to analysis of only the subset of healthier patients that started the study. Nutrition counseling plus oral supplementation improved anorexia and pain in NSCLC patients, although no body composition measurements were reported in this study (74). Finally, treatment of lung cancer patients with nabilone, a synthetic analog of THC, led to decreased pain and increased carbohydrate intake, but there was no improvement in anorexia or BMI (75).

## Conclusions and future directions

This review provides an overview of cancer cachexia pathophysiology including contributors to negative energy balance, the common link of inflammation between cancer cachexia and cancer-related pain, and evidence for an association between cachexia and pain in cancer patients. Although existing literature presents significant evidence that cancer pain and cachexia often occur together, causal relationships cannot be established from these observational studies. The simultaneous presence of both cancer pain and cachexia may simply be evidence of advancing disease. However, pain may also contribute to cachexia by decreasing appetite, food intake, and the ability to be physically active. Increased awareness of the importance of cachexia management on clinical outcomes in cancer patients has led to the development of cachexia clinics with multidisciplinary care to treat nutrition impact symptoms. Pain is one factor to address in these settings. To further understand the interplay between pain and cachexia, prospective clinical studies examining how pain management in cachectic patients affects appetite, food intake, physical functioning, body weight and composition, and clinical outcomes related to cancer are needed.

## Author contributions

The author confirms being the sole contributor of this work and has approved it for publication.

## Conflict of interest

The author declares that the research was conducted in the absence of any commercial or financial relationships that could be construed as a potential conflict of interest.

## Publisher's note

All claims expressed in this article are solely those of the authors and do not necessarily represent those of their affiliated organizations, or those of the publisher, the editors and the reviewers. Any product that may be evaluated in this article, or claim that may be made by its manufacturer, is not guaranteed or endorsed by the publisher.
